# Survival comparison between radical surgery and definitive chemoradiation in 267 esophageal squamous cell carcinomas in a single institution: A propensity-matched study

**DOI:** 10.1371/journal.pone.0177133

**Published:** 2017-05-09

**Authors:** Hideomi Yamashita, Yasuyuki Seto, Ryousuke Takenaka, Kae Okuma, Tomoki Kiritooshi, Kazuhiko Mori, Kazuhiko Yamada, Takashi Fukuda, Michio Kaminishi, Osamu Abe, Keiichi Nakagawa

**Affiliations:** 1 Department of Radiology, University of Tokyo Hospital, Tokyo, Japan; 2 Department of Gastrointestinal Surgery, University of Tokyo Hospital, Tokyo, Japan; 3 Department of Gastrointestinal Surgery, Mitui Memorial Hospital, Tokyo, Japan; 4 Department of Gastrointestinal Surgery, National Center for Global Health and Medicine, Tokyo, Japan; 5 Department of Gastrointestinal Surgery, Saitama Cancer Center, Saitama, Japan; 6 Department of Gastrointestinal Surgery, Showa General Hospital, Tokyo, Japan; Baylor College of Medicine, UNITED STATES

## Abstract

**Objective:**

To compare radical surgery with definitive chemoradiation (CRT) for esophageal squamous cell carcinoma using propensity score (PS) matching at our single institution.

**Materials and methods:**

A total of 386 consecutive, surgically treated and 243 CRT-treated cases between 2001 and 2014 were analyzed. PS was calculated using multivariable analysis (logistic regression) for pairs of variables such as treatment time, age, sex, primary tumor location, clinical stage, and clinical T- and N-stage for patients after excluding clinical T4 and M1 cases. According to PS, 133 surgically-treated and 134 CRT-treated cases were selected randomly by software.

**Results:**

The patients’ median age was 68 years in the CRT group and 71 years in the surgery group. Clinical stage II-III, T3, N0 (according to the 7th American Joint Committee on Cancer-2009), and upper plus middle thoracic esophageal disease were seen in 68%, 44%, 54%, and 59%, respectively, in the CRT group and 64%, 47%, 55%, and 64%, respectively, in the surgery group. The 3- and 5-year overall survival was 47.1% and 34.0% in the CRT group and 68.3% and 54.4% in the surgery group (*p* = 0.0019). The 3- and 5-year progression-free survival was 45.3% and 38.8% in the CRT group and 61.1% and 54.4% in the surgery group (*p* = 0.022).

**Conclusion:**

CRT may be inferior to surgery in survival, although a selection bias for patients selected for a non-operative approach cannot be excluded, especially since surgery is the standard of care at this institution. A prospective randomized clinical trial will be necessary to draw a definite conclusion.

## Introduction

The esophageal cancer is generally known for its very poor prognosis [1–3]. Esophagectomy for the purpose of radical cure remains the most effective therapy for the treatment of locally advanced esophageal cancer. Recent reports on esophagectomy showed a great improvement in the perioperative outcomes and survival achieved [4]. Although the 3-year overall survival (OS) rate after treatment with aggressive resection alone for esophageal cancer remains less than 35%, it has improved up to around 60% after treatment with aggressive resection with/without perioperative chemotherapy [2–3].

In recent years, oncologists have suggested that a non-resected treatment modality by use of definitive combined chemoradiation therapy (CRT) can become a standard of care for locally advanced esophageal cancer [5–8]. Though surgery alone or CRT have generally been accepted as reasonable options for patients with locoregional esophageal cancer, the 5-year survival rate with either management is approximately 20–30% [9–10]. It is unconcluded whether definitive CRT can deliver treatment outcomes equivalent to those of surgery, because there is only one small-sized prospective randomized trial comparing definitive CRT and radical esophagectomy [11]. It is extremely difficult to conduct a clinical randomized trial due to a big difference in a feature of treatment and side effects. Patients with resectable esophageal cancer of stages II-III were requested to choose between definitive CRT and radical surgery in the way of their primary therapy. This is a retrospective study seeking to compare radical surgery with definitive CRT for esophageal squamous cell carcinoma using propensity score (PS) matching.

## Materials and methods

**Fig 1:**
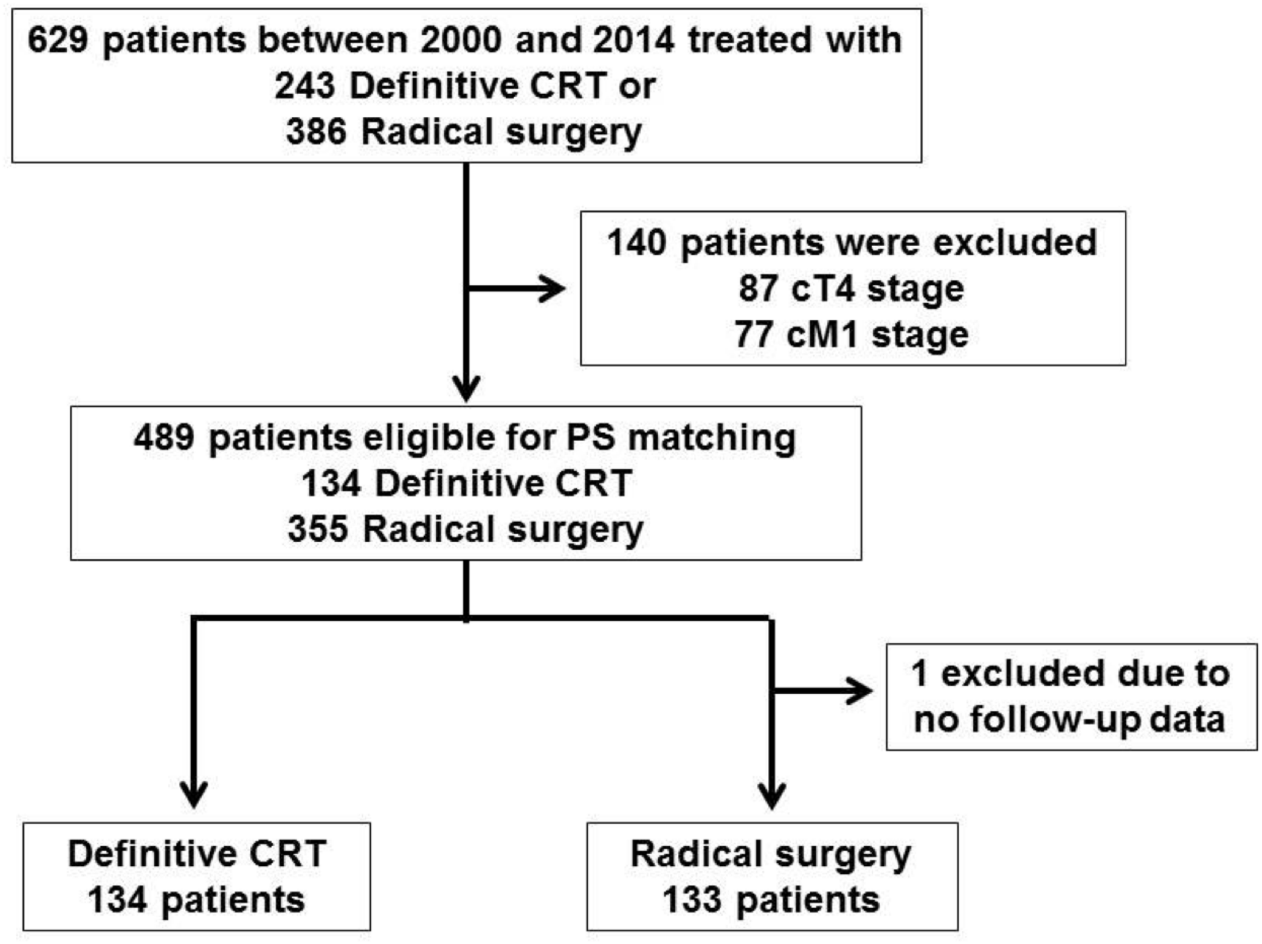
Consort diagram of the study.

Between June 2000 and December 2014, 629 consecutive patients diagnosed with esophageal cancer in stages I-IV were treated with definitive intent at the University of Tokyo Hospital (S1 Table). The patients were ineligible for endoscopic mucosal resection or endoscopic submucosal dissection. A total of 243 patients were treated with CRT and 386 with surgery and were surveyed retrospectively for this study (Fig 1). Each esophageal carcinoma was staged according to the 7th American Joint Committee on Cancer TNM clinical stage classification (2009) using computed tomography scan and endoscopic ultrasound with/without magnifying endoscopy or FDG-PET scan. The last follow-up was performed in October 2015. Patients with clinical T4 (87 cases) and/or M1 (77 cases) were excluded. As a result, 489 patients were included in this study; 134 were treated with CRT and 355 with surgery (S1, S2 and S3 Tables). Patients judged as having technically unresectable cancer, patients who refused to undergo surgery, or those considered medically unfit for surgery were eligible for definitive CRT. This study has been done retrospectively and was approved by a local ethic/IRB board (No. 3372).

From June 2000 to December 2014, 629 consecutive patients diagnosed with esophageal cancer in all stages were treated for the purpose of cure at the University of Tokyo Hospital. The patients were not considered an indication for endoscopic mucosal resection (EMR) or endoscopic submucosal dissection (ESD). A total of 243 patients were treated with definitive CRT and 386 with radical surgery as initial main therapy. They were taken a retrospective survey for this study (Fig 1). The staging of each esophageal carcinoma was according to the 7th American Joint Committee on Cancer TNM clinical stage classification (2009) with the use of computed tomography (CT) scan and endoscopic ultrasound (EUS) with/without magnifying endoscopy or FDG-PET scan. The final follow-up was conducted in October 2015. Patients with clinical T4 stage of 87 cases and/or M1 stage of 77 cases were eliminated. Accordingly, 489 patients (78%) were registered in this study; 134 (27%) were treated with definitive CRT and 355 (72%) with radical surgery. Patients with technically non-resectable lesion, patients who rejected to have surgery, or patients who were considered medically unsuitable for radical surgery were treated with definitive CRT. This retrospective study was approved by a local ethic/IRB board (No. 3372).

### Radiotherapy planning

From 2000 to 2011, the whole thoracic esophageal irradiation was employed in all cases other than old-age patients, who underwent involved-field radiotherapy (IFRT). After February 2011, we launched a prospective study regarding IFRT, and since then IFRT has been used in all cases. All patients in both ENI and IFRT were treated with a total RT dose of 50–50.4 Gy delivered in 1.8–2 Gy per fraction over 5–5.6 weeks. In the ENI arm, the clinical target volume (CTV) was defined as the whole thoracic esophagus (= from the supraclavicular fossae to the esophago-gastric junction) including the gross tumor volume (GTV) plus a 5 mm margin. In the IFRT arm, computed tomography (CT) and/or PET and endoscopic extension were used to define GTV for each patient. All LNs with a diameter at least one cm in short axis in CT or positive by ^18^FDG-PET was included in the GTV. The CTV was generated by using no radial margin and 2 cm longitudinal margins to the GTV-primary, and by using no margins for the GTV-LNs. The planning target volume (PTV) was then generated by applying a 5 mm radial margin and a 10 mm longitudinal margin to the CTV. Depending on the lesion in some cases, PTV was sometimes split and the irradiated fields were divided into separate parts.

### Chemotherapy regimen

In the CDDP group, treatment consisted of 2–4 courses of chemotherapy every 3 or 4 weeks by 5-fluorouracil (5-FU) (1,000 mg/m^2^/24 h by continuous infusion from days 1–4) and cisplatin (CDDP) (75 mg/m^2^ in a bolus infusion on day 1); standard techniques were used for hydration and alkalization [12].

When patient’s renal or cardiac functions were slightly bad such as estimate glomerular filtration rate was 45–60 or patient had a medical history of heart disease, we gave nedaplatin (NDP) in place of CDDP, because NDP has less renal toxicity than CDDP, and the vigorous hydration needed to safely protect against cisplatin-related nephrotoxicity might affect cardiac function. These patients were thought to be able to receive surgery since chemotherapy could not be administered for inoperable patients with poor renal or cardiac function. In these cases, chemotherapy consisted of two cycles of 5-FU (800 mg/m^2^/day, days 1–4 and days 29–32, continuous) combined with NDP (80 mg/m^2^, day 1 and day 29, bolus) [13].

### Surgery

Patients received thoracotomy as radical resection of subtotal thoracic esophagectomy in addition to regional lymphadenectomy. No patient had transhiatal esophagectomy. Regional lymph nodes included perigastric nodes as well as mediastinal, and for this reason regional lymphadenectomy meant not less than a two-field lymphadenectomy. Esophageal reconstruction was performed by use of the stomach, colon, or jejunum. Postoperative adjuvant chemotherapy was recommended in patients with pathologically confirmed lymph node metastasis and who were free of postoperative severe adverse effects. These patients were given two courses of postoperative CF regimen (cisplatin, 80 mg/m^2^, day 1; 5-FU, 800 mg/m^2^/day, days 1–5) at intervals of 4 weeks.

### Follow-up schedule

In the CRT group, the follow-up way was that iodinated contrast-enhanced CT scan at the 2nd week after the end of RT and upper gastrointestinal endoscopy plus biopsy from the primary tumor location at the 4th week after the end of RT and followed by CT scans at intervals of 3–4 months for the first 3 years, 4–6 months until 5 years, and 6–12 months afterward were performed. In the surgery group, CT scans were conducted at intervals of 3–4 months for the first 3 years, 4–6 months until 5 years, and 6–12 months afterward. When residual tumor and/or relapse were suspected, FDG-PET scan was added if at all possible.

### Statistical analysis

The PS represents the relationship between multiple characteristics and receipt of treatment. Matching on the PS can create a covariate balance between the treatment (definitive CRT) and control (radical surgery) groups to reduce confounding by indication [14, 15]. The PS was calculated by using a logistic regression model, with the treatment of interest as the outcome measure. By using the PS-matched cohort, the effect of different clinicopathologic factors on survival was assessed. Variables that are considered for PM were entered into the fully adjusted Cox proportional hazards model based on statistical (*p* < 0.05) or clinical (treatment time, age, sex, primary tumor location, clinical stage, clinical T-, N-, and M-stage) significance for 386 surgery and 230 CRT cases between 2001 and 2014. The software that we used was R. Additionally, we performed the 1:1 PS matching in Stage I patients and analyzed survivals among Stage I patients. The same was also done in stage II and III patients.

Comparisons of patient and tumor characteristics, toxicity, and site of first failure were performed with χ2 tests, 2-sample t-tests or Wilcoxon Rank Sum tests. The Kaplan-Meier method was used to estimate survival data. The distribution of survival time between arms was tested by the log-rank method. Student’s t-test was used for comparison of means. Fisher’s exact test was used for comparisons of categorical data. All *p* values were based on a 2-sided test, and the differences were regarded as statistically significant when *p* < 0.05.

## Results

### PS matching

PS was calculated using binary logistic regression analysis. Odds ratios for the CRT and surgery groups were 6.57 in c-stage (*p* < 0.0001), 3.60 in c-N stage (*p* = 0.0006), and 2.25 in c-T stage (*p* = 0.039). According to PS, 267 patients were left randomly by software, of whom 134 were treated with CRT and 133 with surgery (S5 Table). One patient in the surgery group was excluded because of insufficient survival data. Patient and tumor characteristics both before and after PS matching are shown in Table 1. There were no differences in primary tumor location (*p* = 0.72), sex (*p* = 0.23), age (*p* = 0.99), c-stage (*p* = 0.71), c-T stage (*p* = 0.44), and c-N stage (*p* = 0.85) between the two groups, although in the surgery group significantly younger patients were included than in the CRT group before PS matching (*p* < 0.0001). Since Eastern Cooperative Oncology Group performance status (ECOG PS) was either 0 or 1 in all patients in the both groups, it was not included for PS matching. ECOG PS was 0 or 1 in 3.7% and 96.3% out of 134 patients in the CRT group and in 3.9% and 96.1% out of 355 patients in the surgery group, respectively, since in our institution only patients whose ECOG PS is either 0 or 1 are a candidate for radical surgery or definitive CRT. In surgery group, 56 patients out of 133 were treated with neoadjuvant (16 patients) and/or adjuvant treatment (44 patients). In definitive CRT group, 23 patients (17%) were given 50Gy in 25 fractionations and 111 patients (83%) were 50.4Gy in 28 fractionations.

**Table 1:** Characteristics before and after propensity score matching.

Factors	Before PS matching		CRT		p value by χ^2^ test	After matching		p value by χ^2^ test
	Surgery					Surgery		
	N	%	N	%		N	%	
Total	355		134			133		
Location					0.48			0.72
Ce	11	3.1	6	4.5		5	3.8	
Ut	42	11.8	21	15.7		18	13.5	
Mt	143	40.3	58	43.3		67	50.4	
Lt	149	42.0	49	36.6		43	32.3	
Sex					0.23			0.23
Male	300	84.5	116	86.6		111	83.5	
Female	55	15.5	18	13.4		22	16.5	
Age (y.o.)								
-59	94	26.5	18	13.4	0.0022	9	6.8	0.071
60–69	138	38.9	54	40.3	0.77	34	25.6	0.010
70–79	105	29.6	42	31.3	0.70	75	56.4	<0.0001
80-	18	5.1	20	14.9	0.0003	15	11.3	0.38
Median	66 (39–92)		68 (44–86)		<0.0001*	71 (39–85)		0.99
c-Stage					0.50			0.71
I	99	27.9	43	32.1		48	36.1	
II	144	40.6	47	35.1		41	30.8	
III	112	31.5	44	32.8		44	33.1	
c-T stage					0.28			0.44
T1	142	40.0	48	35.8		52	39.1	
T2	51	14.4	27	20.1		19	14.3	
T3	162	45.6	59	44.0		62	46.6	
c-N stage					0.19			0.85
N0	167	47.0	72	53.7		73	54.9	
N1	188	53.0	62	46.3		60	45.1	

*: t-test

Abbreviation: CRT = chemoradiation, Ce = cervical, Ut = upper thoracic, Mt = middle thoracic, Lt = lower thoracic, PS = propensity score

### Survival

**Fig 2:**
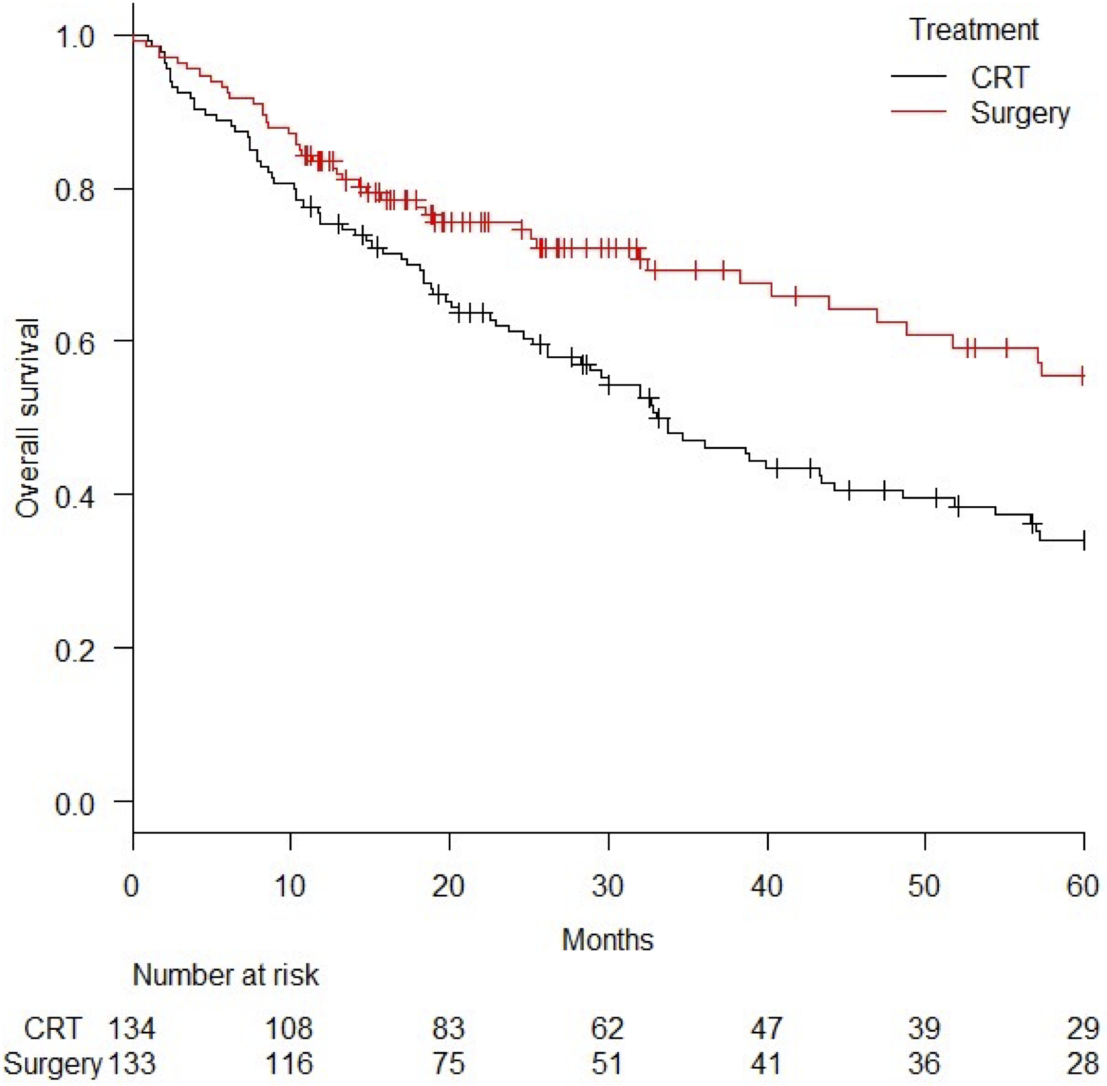
Comparison of overall survival curves for surgery and chemoradiation (CRT) after propensity score matching. *Abbreviation*; CRT = chemoradiation.

OS curves for the surgery (n = 133) and salvage groups (n = 134) after PS matching are shown in Fig 2. The 3-year and 5-year OS was 68.3% (95%CI = 58.5–76.3%) and 54.4% (95%CI = 42.6–64.8%), respectively, in the surgery group and 47.1% (95%CI = 38.1–55.7%) and 34.0% (95%CI = 25.4–42.9%), respectively, in the CRT group (log-rank *p* value = 0.0019).

**Fig 3:**
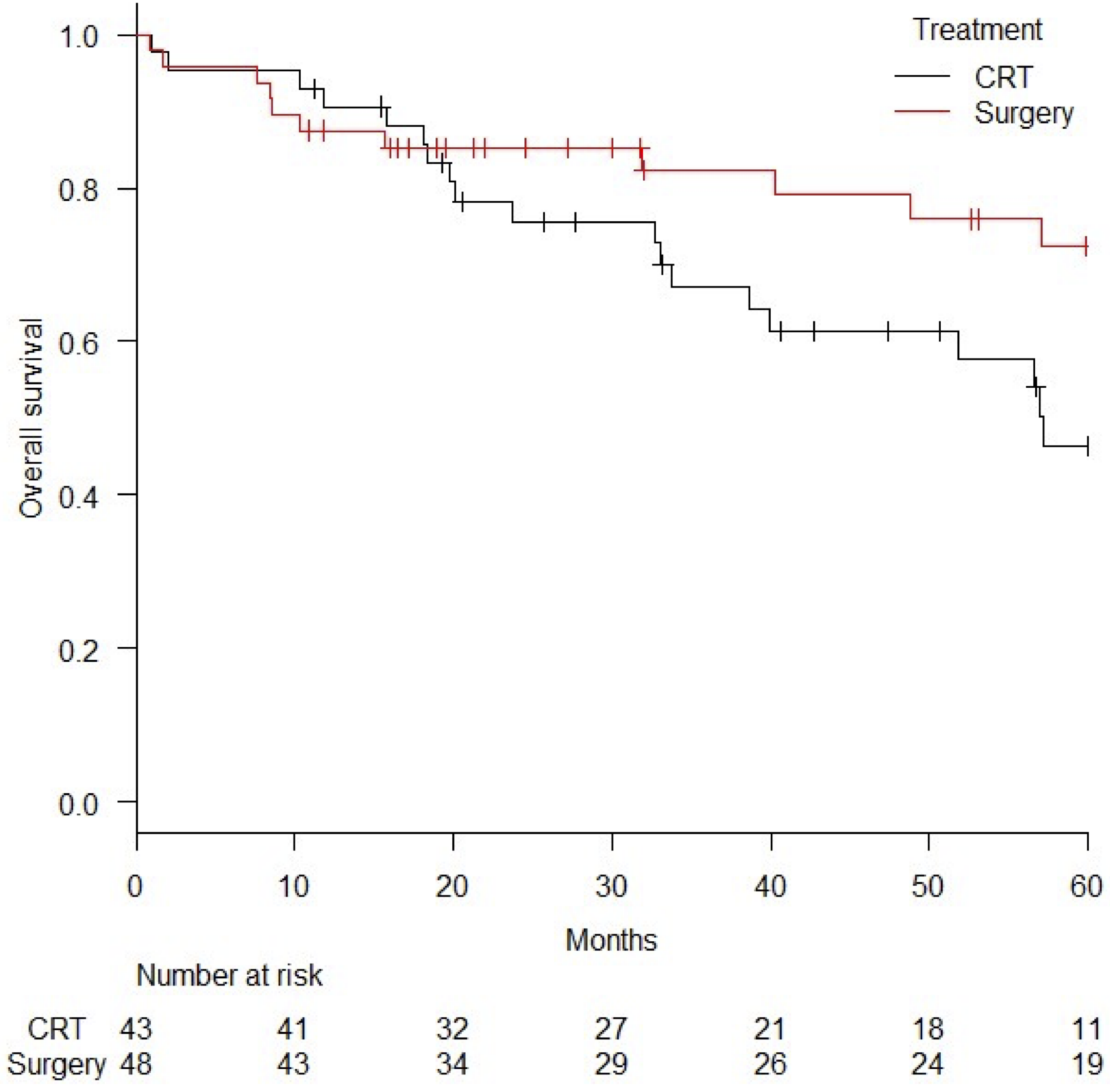
Comparison of progression-free survival curves for surgery and chemoradiation (CRT) after propensity score matching. *Abbreviation*; CRT = chemoradiation.

Progression-free survival (PFS) curves for the surgery (n = 133) and CRT groups (n = 134) after PS matching are shown in Fig 3. The 3-year and 5-year PFSs were 61.1% (95%CI = 51.5–69.4%) and 54.4% (95%CI = 43.8–63.9%), respectively, in the surgery group and 45.3% (95%CI = 36.2–53.9%) and 38.8% (95%CI = 29.2–48.4%), respectively, in the CRT group (log-rank *p* value = 0.022).

**Fig 4:**
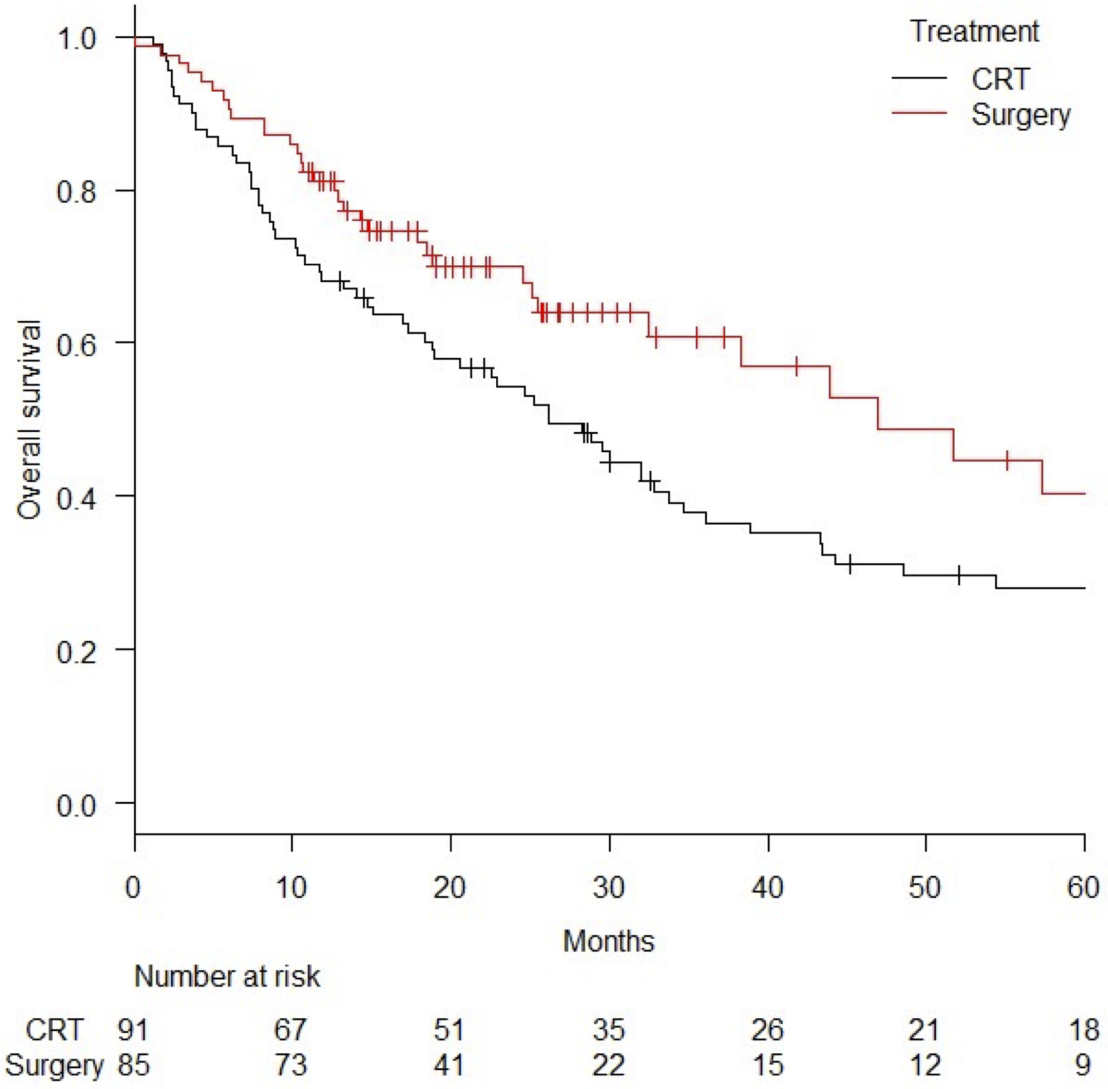
Comparison of overall survival curves for surgery and chemoradiation (CRT) in stage I disease. *Abbreviation*; CRT = chemoradiation.

In limiting the analysis to 91 cases with stage I disease, the 3-year and 5-year OS was 82.8% (95%CI = 67.2–91.4%) and 75.2% (95%CI = 57.0–86.6%), respectively, in the surgery group and 66.1% (95%CI = 49.9–78.1%) and 44.5% (95%CI = 27.9–59.8%), respectively, in the CRT group (log-rank *p* value = 0.018) (Fig 4). The 3-year and 5-year PFS was 82.3% (95%CI = 67.3–90.8%) and 72.5% (95%CI = 55.0–84.1%), respectively, in the surgery group and 57.8% (95%CI = 40.0–72.0%) and 39.2% (95%CI = 21.0–57.0%), respectively, in the CRT group (log-rank *p* value = 0.0078). Five patients with stage I disease died of other cancers such as tongue cancer in two patients, hypopharyngeal cancer in one, primary lung cancer in one, or hepatocellular carcinoma in one patient. Only five patients died of esophageal cancer recurrence, and 11 patients died from reasons other than cancer: Rupture of a thoracic arterial aneurysm, community-acquired pneumonia, respectively, in two patients, cardiac failure in two patients, aspergillus pneumonia, perforation of intestinal tract after large bowel obstruction from diaphragmatic hernia, renal failure, respiratory failure, or aspiration pneumonia, each in one patient.

**Fig 5:**
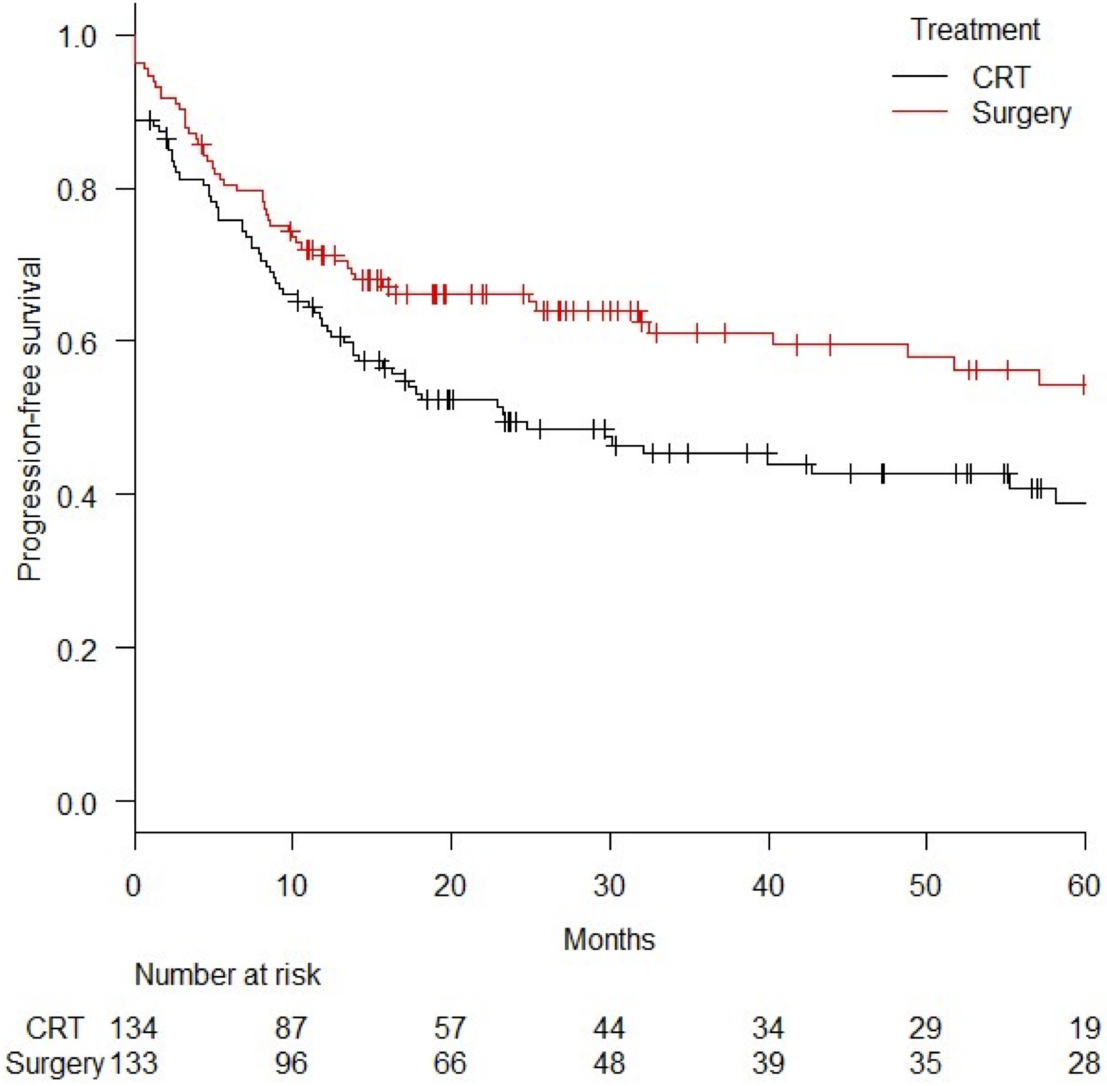
Comparison of overall survival curves for surgery and chemoradiation (CRT) in stages II-III disease. *Abbreviation*; CRT = chemoradiation.

In stages II-III, the MST was 46.9 months in the surgery group (n = 85) and 26.2 months in the CRT group (n = 91) (log-rank *p* value = 0.024). The 3-year and 5-year OS were 60.7% (95%CI = 47.2–71.8%) and 40.3% (95%CI = 23.7–56.3%), respectively, in the surgery group and 37.9% (95%CI = 27.5–48.1%) and 28.1% (95%CI = 18.7–38.3%), respectively, in the CRT group (Fig 5). The 3-year and 5-year PFS was 48.3% (95%CI = 35.8–59.7%) and 43.9% (95%CI = 30.0–56.9%), respectively, in the surgery group and 26.2% (95%CI = 17.3–35.9%) and 21.2% (95%CI = 12.8–30.9%), respectively, in the CRT group (log-rank *p* value = 0.0046).

The PS matching was performed again in stage I patients and 40 patients in each group were analyzed. The 3-year and 5-year OS among stage I patients were 83.7% and 74.4% in the surgery group versus 69.7% and 51.2% in the CRT group (*p* = 0.032). The 3-year and 5-year PFS among stage I patients were 86.3% and 79.5% in the surgery group versus 89.0% and 80.9% in the CRT group (*p* = 0.69). The PS matching was performed in stage II-III patients and 82 patients in each group were analyzed. The 3-year and 5-year OS among stage II-III patients were 61.2% and 47.8% in the surgery group versus 36.3% and 28.4% in the CRT group (*p* = 0.0042). The 3-year and 5-year PFS among stage II-III patients were 55.3% and 53.0% in the surgery group versus 25.8% and 20.3% in the CRT group (*p* = 0.000024).

### Recurrent site

Persistent tumor was seen in 7 patients after definitive CRT. Microscopic and macroscopic residual tumor was seen in one and two patients after radical surgery, respectively. Recurrence was seen in 33 patients after radical surgery and in 58 patients after definitive CRT. As to the first recurrent site after radical surgery, distant metastasis was seen in 21 patients and local recurrence in 12 patients. As to the first recurrent site after definitive CRT, distant metastasis alone was seen in 21 patients, local recurrence alone in 28 patients and both distant and local recurrence in 9 patients.

### Salvage treatment

Salvage surgery was performed in 2 patients with persistent tumor and in 10 patients with loco-regional recurrent lesions after definitive CRT. On the other hand, salvage CRT with curative intent and palliative RT-alone were performed in 15 patients and 2 patients, respectively, with recurrent lesions after radical surgery. Palliative chemotherapy was performed in 5 patients as initial therapy for recurrent sites after radical surgery and in 35 patients after definitive CRT.

### Adverse events after radical surgery

Grade 2 adverse events were seen in 17 patients (12.8%), grade 3 in 44 patients (33.1%), and grade 4 in 19 patients (14.3%) after surgery. Recurrent laryngeal nerve dysfunction (31 cases), anastomotic leaks (31 cases), and pulmonary complications (28 cases) were seen at a high frequency.

### Adverse events after definitive CRT

As to hematological adverse events grade 3 and 4 during CRT, leukopenia were seen in 78 patients (58.2%) and 19 patients (14.2%), respectively, anemia in 29 patients (21.6%) and 0 patient, respectively, and thrombocytopenia in 43 patients (32.1%) and 24 patients (17.9%), respectively. On the other hand, non-hematological side effects, grade 3 and 4 were seen in 19 patients (14.2%) and 9 patients (6.7%), respectively. Radiation esophagitis was seen at a high frequency. Only one patient succumbed to treatment-related death by esophageal bleeding at 2.1 months after starting definitive CRT.

## Discussion

Both OS and PFS in the CRT group were significantly worse than those in the surgery group despite exclusion of T4 or stage IV cases that did not fulfill the requirements for radical surgery. Although background factors such as age and staging were uniformed by PS matching, the selection bias would be left. In our institution, salvage CRT was aggressively performed for postoperative recurrent cases, and salvage surgery was performed for recurrent cases after definitive CRT.

We described OS and PFS for each stage I and II-III of 267 patients after PS matching. In the present study, we thought that the reason why definitive CRT was significantly worse than radical surgery may be that the result of stage I in the surgery group was much better than that in the CRT group and the result of stage II-III may be similar in both groups. If that was the case, we were supposed to perform PS matching after focused on only stage II-III one more time, like the previous study [16].

The results of surgery may be too optimistic. The 3-year and 5-year OS in our study were 68% and 54%, respectively. According to cancer statistics in Japan concerning clinical outcome after surgical resection, 3-year- and 5-year OS were 85% and 74%, respectively, in stage I, 64% and 50%, respectively, in stage II, and 39% and 31%, respectively, in stage III [17]. A study by the Japan Clinical Oncology Group (JCOG) 9907 [18], compared preoperative neoadjuvant CT with postoperative adjuvant CT using cisplatin plus 5-fluorouracil for clinical stage II/III (except T4 tumor) squamous cell carcinoma of the thoracic esophagus (n = 330); the 3-year OS rate was 63% in the neoadjuvant CT group versus 48% in the postoperative CT group (*p* = 0.0014). When considering the results from Western countries, there are various obstacles to interpreting the findings in relation to practice in Japan, as there are great differences in modes of surgical resections and survival results between Western countries and Japan as well as differences in tumor cell biology and incidence of squamous cell carcinoma and adenocarcinoma. In Japan, radical surgery with extensive nodal dissection is commonly indicated, and most tumors are squamous cell carcinomas.

In the JCOG 9204 study of a multicenter randomized controlled trial [19], the 5-year disease-free survival rate was 45% with surgery alone for esophageal squamous cell carcinoma undergoing radical surgery, and 55% with surgery plus postoperative adjuvant chemotherapy (*p* = 0.037) and the 5-year OS rate was 52% and 61%, respectively (*p* = 0.13). Risk reduction by postoperative chemotherapy was remarkable in the subgroup with lymph node metastasis. In our institution, surgery alone, although it was not standard treatment now, had been performed for patients with early stage and/or without lymph node metastasis, too.

The 2-year-, 3-year-, and 5-year OS were 61%, 47%, and 34%, respectively, and the MST was 33.0 months in our study. On the other hand, in the previous large-scale randomized clinical trial, RTOG 85–01, of definitive CRT for esophageal cancer, the 5-year OS was 26% and the MST was 13 months [5, 6] and the 2-year OS was 40% and the MST was 18 months in INT 0123 [12]. A phase II trial of JCOG 9906 on definitive CRT for stages II and III with the exception of T4 tumor [20] reported 45% of the 3-year overall survival. The clinical result of the CRT group in the present study was comparable to that of those reports [5, 6, 12, 20]. Other papers [21,22] with similar design and primary outcomes to the present study in this field reported the slightly higher survival percentage than ours (Table 2).

**Table 2:** Comparing our results to the other studies existing today for esophageal squamous cell carcinoma.

First author	Treatment method	No.	Stage	3-y OS	5-y OS
Matsuda S [21] 2015 Retrospective	TTE	112	I: 26%	71%	58%
			II: 24%	IA: 90%	IA: 90%
			III: 41%	non-IA: 67%	non-IA: 51%
			IV: 9%		
	dCRT	65	I: 35%	62%	58%
			II: 23%	IA: 94%	IA: 87%
			III: 31%	non-IA: 50%	non-IA: 43%
			IV: 11%		
Nomura M [22] 2016 PS analysis	NAC-S	206	I: 8%	67%	57%
			II: 27%	I: 100%	I: 80%
			III: 56%	II: 81%	II: 77%
			IV: 9%	III: 57%	III: 46%
				IV: 56%	IV: 34%
	CRT	200	I: 28%	59%	50%
			II: 22%	I: 89%	I: 76%
			III: 35%	II: 77%	II: 75%
			IV: 15%	III: 29%	III: 18%
				IV: 34%	IV: 29%
Our data PS analysis	Surgery	133	I: 36%	68%	54%
			II: 31%	I: 83%	I: 75%
			III: 33%	II-III: 61%	II-III: 40%
	CRT	134	I: 32%	47%	34%
			II: 35%	I: 66%	I: 45%
			III: 33%	II-III: 38%	II-III: 28%

*Abbreviation*: OS = overall survival, TTE = transthoracic esophagectomy, dCRT = definitive chemoradiotherapy, PS = propensity score, NAC-S = neoadjuvant chemotherapy followed by surgery, CRT = chemoradiotherapy

According to phase II JCOG 9708 trial which reported the efficacy of definitive CRT for 72 patients with Stage I esophageal squamous cell carcinoma, 4-year OS and PFS were 80.5% (95% CI: 71.3–89.7%) and 68.1% (95% CI: 57.3–78.8%), respectively [23]. The relatively worse survival after CRT of 60% in the 4-year OS in the present study was seen than in JCOG 9708 trial. This reason seems that 16 out of 43 patients (37%) with stage I after definitive CRT died of other diseases than esophageal cancer.

In our center, as was so often the case in many Japanese institutions, the patients who received definitive CRT were either technically non-resectable, rejected surgery, or were unfit to surgery due to medical disorder. The choice of treatment for esophageal cancer was argued at a biweekly multidisciplinary cancer board.

The most appropriate management of esophageal cancer is still inconclusive. Locally advanced esophageal cancer usually has a poor prognosis due to high rate of local recurrence in spite of very intense management of radical surgery with or without postoperative adjuvant chemotherapy [1, 2]. According to the National Comprehensive Cancer Network (NCCN) practice guidelines, either preoperative CRT, definitive CRT with doses of 50–50.4 Gy only for patients who decline surgery, or esophagectomy are considered as standard treatments in esophageal squamous cell carcinoma [24]. In the North American continent, preoperative CRT followed by surgery has been performed with a high frequency in spite of a lack of persuasive data to show its effectiveness [25–30]. A meta-analysis proved a survival benefit in preoperative CRT as compared to surgery monotherapy [31]. However, pleurisy, pericarditis, heart failure, esophagitis, pneumonitis, and so on have possibilities as adverse effects after definitive CRT. According to a Japanese report about 78 patients who had achieved a complete response after definitive CRT, the incidence of grades 3 and 4 of those toxicities were 10.3% and 3.8%, respectively [32]. Irradiation of the whole esophagus, independent of the tumor stage, was too extensive, and this was probably the main reason for the high frequency of esophagitis in the present study.

In an effort to reduce the gap between the results of definitive CRT and surgery, the salvage surgery for local residual lesion right after definitive CRT is actively performed in our institution. The frequency of salvage surgery that was conducted after definitive CRT was 15% in the JCOG 9906 study [20] and 6 out of 36 cases (17%) in the CURE study [11]. In our study, 12 patients out of 134 patients (9.0%) in the CRT group underwent salvage surgery.

The limitation of this study is a potential sample selection bias. Several potential biases may have affected the results. First, the criteria adopted for the choice of treatment (definitive CRT vs. radical surgery) were: “Patients who rejected surgery or those judged unfit for surgery by concurrent medical illness were eligible for definitive CRT”. Secondly, there is a possibility that comorbidities were not equalized well in the both groups. In addition to the long duration of the study, other potential biases are the heterogeneities in radiotherapy methods and chemotherapy cycles.

## Conclusion

CRT may be inferior to surgery in survival, although a selection bias cannot be excluded for patients selected for a non-operative approach especially since surgery is the standard of care at this institution. A prospective randomized clinical trial will be necessary in order to draw a definite conclusion.

## Supporting information

S1 Table 629 patients’ raw data of esophageal cancer in stages I-IV treated with definitive intent. (XLSX)

S2 Table 489 patients’ raw data after excluding clinical T4 and/or M1 cases. (XLSX)

S3 Table 134 patients’ raw data in the CRT group after excluding clinical T4 and/or M1 cases. (XLSX)

S4 Table 355 patients’ raw data in the surgery group after excluding clinical T4 and/or M1 cases. (XLSX)

S5 Table 267 patients’ raw data after 1:1 PS matching. (XLSX)

## Abbreviations

5-FU: 5-fluorouracil
CRT: chemoradiation
CDDP: cisplatin
CTV: clinical target volume
CI: confidence interval
ENI: elective nodal irradiation
GTV: gross tumor volume
IFRT: involved field radiotherapy
JCOG: Japan Clinical Oncology Group
MST: median survival time
NCCN: National Comprehensive Cancer Network
NDP: nedaplatin
NA: not reached
OS: overall survival
PS: propensity score
